# A perspective on Chiropractic Councils on Education accreditation standards and processes from the inside: a narrative description of expert opinion: Part 1: Themes

**DOI:** 10.1186/s12998-019-0275-6

**Published:** 2019-09-12

**Authors:** Stanley I. Innes, Vicki Cope, Charlotte Leboeuf-Yde, Bruce F. Walker

**Affiliations:** 1 College of Science, Health, Engineering and Education, Murdoch University, Murdoch, Australia; 2 Institute for Regional Health Research, University of Southern Denmark, DK-5000 Odense, Denmark

**Keywords:** Accreditation, Chiropractic, Competence, Qualitative

## Abstract

**Background:**

The aim of this study was to report on key informant opinions of Councils on Chiropractic Education (CCE) regarding recent research findings reporting on improving accreditation standards and processes for chiropractic programs (CPs).

**Methods:**

This qualitative study employed in-depth semi-structured interviews with key experienced personnel from the five CCEs in June and July of 2018. The interviews consisted of open-ended questions on a range of issues surrounding accreditation, graduate competency standards and processes. All interviews were audio-recorded, and transcribed verbatim. The transcripts were analysed to develop codes and themes using thematic analysis techniques assisted by NVivo coding software. The study followed the COREQ guidelines for qualitative studies.

**Results:**

Six themes were isolated from the interview transcripts; they were: professional differences; keep it in the family; to focus on outcomes or be prescriptive?; more resources please; inter-profession integration; and CPs making ends meet. Most respondents saw a need for CCEs standards and processes to improve interdisciplinarity while at the same time preserving the ‘uniqueness’ of chiropractic. Additionally, informants viewed CCEs as carrying out their functions with limited resources while simultaneously dealing with vocal disparate interest groups. Diverse views were observed on how CCEs should go about their business of assessing chiropractic programs for accreditation and re-accreditation.

**Conclusions:**

An overarching confounder for positive changes in CCE accreditation standards and processes is the inability to clearly define basic and fundamental terms such as ‘chiropractic’ and its resultant scope of practice. This is said to be because of vocal, diverse and disparate interest groups within the chiropractic profession. Silence or nebulous definitions negotiated in order to allow a diversity of chiropractic practice to co-exist, appears to have complicated and hindered the activities of CCEs. Recommendations are made including an adoption of an evidence-based approach to accreditation standards and processes and the use of expertise from other health professions. Further, the focus of attention should be moved away from professional interests and toward that of protection of the public and the patient.

**Electronic supplementary material:**

The online version of this article (10.1186/s12998-019-0275-6) contains supplementary material, which is available to authorized users.

## Background

It is well known that members of the medical profession generally are wary of the chiropractic profession [1]. In our experience, many chiropractors think this is based on an unfair type of bias. Chiropractic is ‘officially’ presented as an evidence-based friendly health care profession, keen to be integrated with the rest of the health care community in the treatment of musculoskeletal conditions [2]. However, the practice of chiropractic is not above criticism. Varied sections of the profession, depending on the part of the world they practice in, promote the use of chiropractic manipulation for a wide range of non-musculoskeletal conditions, which is controversial and evidence of this can easily be seen by reading the self-promotion of chiropractic clinics on their websites from all over the world.

It is therefore not surprising that as the profession has become larger and more visible there have been organised reactions to such practice. For example, recently the Spanish Ministries of Health and Science have announced an intention to implement a ‘Health Protection Plan against Psuedotherapies’ and appears likely to include chiropractic [3]. Attempts to establish university-based education in chiropractic have been rejected in Florida, U.S.A. [4] partially due to resistance from vocal interest groups highlighting unsupported claims by chiropractors and, in Sweden, [5] because of the Universities becoming aware of inappropriate website claims. This has led to the Universities management judging the profession unsuitable to be associated with. In the United Kingdom a systematic campaign of notification to the General Chiropractic Council was undertaken by a group of activists who targeted the inappropriate use of science, to highlight many sub-standard chiropractic websites [6]. In Australia, a movement called ‘Friends of Science’ used chiropractic education as an example of non-evidence based alternative medicine that should be excluded from university education, in part based on the discovery of the opening of a ‘paediatric clinic’ treating some non-musculoskeletal illnesses at the RMIT University chiropractic school clinic in Melbourne, Australia [7–9].

There are other examples, so this leads us to the conclusion that the criticisms are thus not wholly unfounded. Importantly, these aberrant actions by some of the profession have implications for patient safety and quality of care [10–17]. According to a Canadian study, approximately 18% of the practicing chiropractors were found to have practice patterns that could be described as ‘unorthodox’ (*vitalist)* and demonstrated high levels of anti-vaccination attitudes, use of non-evidence based treatment choices, non-guideline use of X-rays, and low levels of inter-professional collaboration [18]. In Australia, chiropractic students were found to have non-evidence-based healthcare beliefs that were resistant to the educative process [19]. Further recent research has identified personality types that impact negatively on chiropractic students’ clinical decisions [20]. Also, chiropractic students in Australia [21] and France [22] have been shown to be poor at recognising when treatment will make no difference to patient outcomes. This inability to acknowledge the limits of competencies was, in one of the studies, very strongly associated with a conservative belief in the ‘powers’ of the subluxation [22].

Further, ‘unorthodox’ or conservative/vitalistic behaviours patterns in chiropractic practice appear to be ‘sensitive’ issues and practitioners are reluctant to participate in studies exploring these issues. A recent study was thwarted through a very low response rate where few chiropractors responded to the ‘sensitive’ questions [23].

The education of chiropractors is one important nexus that should be influencing standards of chiropractic practice. The guardians of these standards are various Councils on Chiropractic Education (CCEs), one in Europe [24], one in Australia [25], one in Canada [26] and one in the United States of America [27], and an international organisation initially formed by these four CCE, the CCE-International [28]. These councils are responsible for accrediting the chiropractic courses in their ‘jurisdiction’ and preferably the standards they set should be homogeneous all over the world. However, there is substantive evidence that the standards they set and monitor are not homogeneous nor of an adequate standard [18, 29–32].

Chiropractic undergraduate institutions issued a declaration in 2015 that chiropractic education programs have an ethical obligation to support an evidence-based teaching and learning environment and global consistency in accreditation and assessment [33]. Thirteen [34] of the 36 chiropractic programs [35] that are accredited by a CCE signed this statement. In 2001, all the regional CCEs became signatories to the CCE-International with the intent to collaborate, assure excellence and consistent quality improvements in chiropractic education through accreditation. However, in 2015 the CCE-USA withdrew from this agreement, without public explanation by the CCE-I or the CCE-USA, and this would appear to considerably reduce the likelihood of agreement between all regions [36]. On the surface this appears to be a failing of CCEs to group together to create and monitor a homogeneous set of high quality standards in the interests of public safety, professional respectability, and workforce portability. Medical education has achieved this [37]. Why has it not happened for chiropractic?

Given there has been a reluctance by some chiropractors to engage in quantitative survey research on these ‘sensitive’ issues, another way needed to be found. Qualitative research is able to explore complex phenomena obtaining an in-depth understanding by seeking the respondents viewpoints on the phenomena of interest [38]. Consequently, we sought to ask people with extensive experience in CCEs, their views on significant matters, in order to provide insights into these questions and concerns from the perspective of those experiencing it. Opinions from experts has proven to be valuable for developing policy [39, 40] and improving educational curricula [41]. To this end, we conducted interviews of experts with first-hand knowledge of the five CCEs.

### Aim

The primary aim of this study was to explore the experience and beliefs of CCE experts on accreditation standards and processes of chiropractic programs (CPs) by seeking their views on:

I. Competencies for graduating chiropractors. In particular the implementation of identical competencies for all CCEs.
II. Accreditation and re-accreditation standards for CPs. In particular the implementation of identical standards for all CCEs.
III. The processes and standards for site inspection teams of CPs.
IV. CCEs monitoring CPs to ensure that students learn important course material.
V. The influence of vitalism and evidence-based practice in CP course material.

This paper addresses the common themes in the responses of the participants across these five issues listed above (Part I). A subsequent paper will be developed exploring the responses to each of these issues in turn, garnering the diverse discussion and controversial professional responses found (Part II).

## Method

This was a qualitative descriptive study utilising in-depth semi-structured interviews in-person via Skype and telephone. The interview questions were generated from recent research that identified a number of issues and concerns with respect to CCE accreditation standards and processes. These questions are summarised in Table 1 and the full interview (*aide de memoir*) is included in Additional file 1.

**Table 1:** Questions asked of CCE experts and the respective study from which it was based on

Question	Study
What are your views about implementing identical graduating chiropractor competency standards for all CCEs?	[29]
Is there anything you would like to change in the domains of competencies for graduating chiropractors?	[29]
What are your views about implementing identical accreditation standards for all CCEs?	[30, 31]
Is there anything you would like to change in the domains of CCE accreditation standards?	[31, 36]
What are your views on the ability of CCE site inspection teams to monitor and improve the quality of CPs?	[31]
What are your views on the CCEs role in CPs to ensure that students learn relevant clinical course material? For example, learning the contra-indications for chiropractic care?	[21]
What are your views on CCEs requiring CPs to teach students about understanding their own personality, attitudes or beliefs and how these may impact on their clinical decisions?	[20]
What are your views about the inclusion of vitalism and evidence based practice into CP course material?	[30, 36]

Ethics approval was obtained from the University Human Research Ethics Committee (2018/055) before recruitment and data collection. The study followed the COREQ guidelines for qualitative studies [42].

### Participants

Thirteen email approaches were made to expert participants. There were 4 non-responders. One non-responder did not answer 3 e-mail approaches. Two non-responders initially agreed but then did not respond to further emails when attempting to arrange an interview time. Another initially agreed, but then expressed concern over a possible conflict of interest with a CCE, and after that did not respond to further email contacts. Finally, one responder missed the Skype interview and provided written responses to the questions. This resulted in 9 key participants (6 men and 3 women) who were interviewed. Two participants were non-chiropractors. The people who serve on the CCE-I are selected from member CCEs. The CCE-I does not have any geographical jurisdiction, nor is it involved in making any accreditation decisions. In order to address this issue, the identified respondents from the CCE-I were also required to have had extensive experience (at least 8 years) with a member agency in these matters.

Characteristics of the sample are not given to protect the anonymity of participants. The nine participants had an average of 14 years’ experience working for a CCE. The interviews were conducted between May and July of 2018 and lasted between 32 and 62 min, with an average duration of 44 min.

Signed or verbal consent was obtained from all participants prior to being interviewed. All transcribed records were kept confidential, with only the investigators having access to the information provided. Participants were de-identified by being assigned a reference number between 1 and 9.

### Participant recruitment

**Fig. 1:**
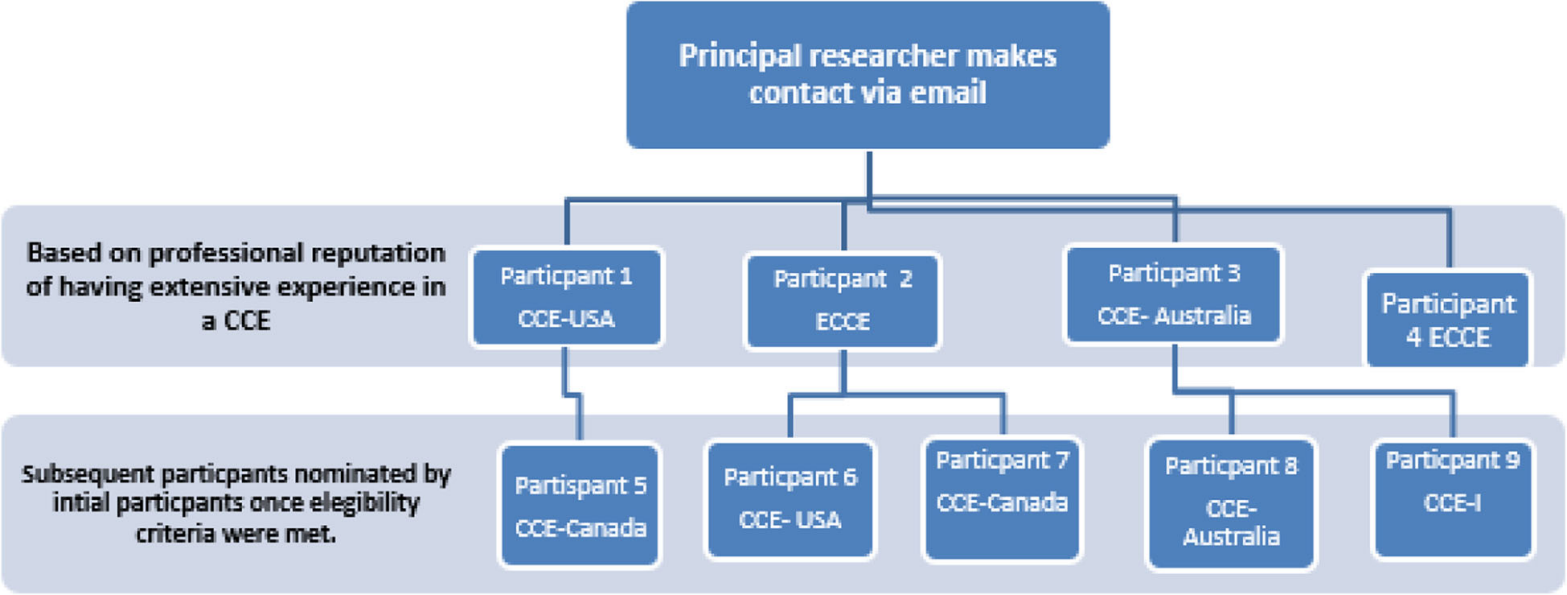
Illustration of snowball sampling

We incorporated snowball sampling. An example of this is shown in Fig. 1. The actual contact details are not provided to protect the confidentiality of the participants. This sampling is a non-probability sampling technique used by researchers to identify potential participants for studies where respondents are hard to locate [43].

Individuals known as having had a long-standing association or had extensive experience with a CCE were contacted via email. The purpose of the study was explained to them and they were asked to identify other potential participants. Two key representatives from each of the 5 CCEs were being sought. CCE participants were expected to have been involved with a Board, Commission or as a site team member for a period of at least 8 years duration. Additionally they were required to be either currently serving, or had done so within the last 5 years and could not have been dismissed from CCE service. As this was to be a narrative description of expert opinions, past research has suggested that 1–2 individuals from each organisation (CCE) is an adequate sample size, that is, this would provide between 5 and 10 individuals in total [44] . Interviews were conducted, transcribed, coded and analysed in-turn. These were reviewed by the lead researcher and then further reviewed and discussed with a qualitative research investigator, as to whether thematic saturation had been reached. The researchers agreed that thematic saturation was reached after the ninth interview. Consequently no further participants were sought.

### Data collection

Data were collected from consenting participants using a semi-structured in-depth interview process. Because the experts were located in North America, Europe, Canada, and throughout Australia or New Zealand, the interviews were to be conducted by either Skype or telephone. One-on-one interviews were held at a time convenient to each participant.

The principle researcher (SI) conducted the interviews (*n* = 9). The nine participants were provided with the pending questions prior to the interview and invited to reflect on them. Participants were also invited to make further comments as they felt appropriate to the topics under discussion during the interview. An *aide de memoire* was used to ensure consistency across all the interviews (Additional file 1). Participant responses were audio recorded on two digital devices and transcribed verbatim.

### Data analysis

All interviews were then imported, organised into themes and analysed using the qualitative analysis NVivo 11 software. Thematic analysis of the recordings was used to analyse the data as outlined by Braun and Clarke [45]. Repeated readings results in familiarisation of the data and leads to identification of recurrent patterns and themes. Using NVivo assistance software and manual coding key, concepts were isolated, and themes and sub-themes were identified.

Trustworthiness of data and interpretation of the study involved four categories: credibility, transferability, dependability and confirmability [46]. To increase credibility, the transcriptions were returned to the interviewees for verification of accuracy. This ensured verification of data. The interviewer was familiar with relevant CCE documentation and this helped ensure credible interpretation of the interactions with the participants, thus improving methodological rigour [47]. To attain dependability and confirmability of the data, the analysis process was reviewed by another qualitative expert (VC).

## Results

### Findings / recurring themes

Nine participants were interviewed. There were six recurring themes raised by the majority of respondents across all questions in the interviews and were considered to be overarching. The six themes were: Professional differences; keep it in the family; to focus on outcomes or be prescriptive?; more resources please; inter-professional integration; and CPs making ends meet.

**Fig. 2:**
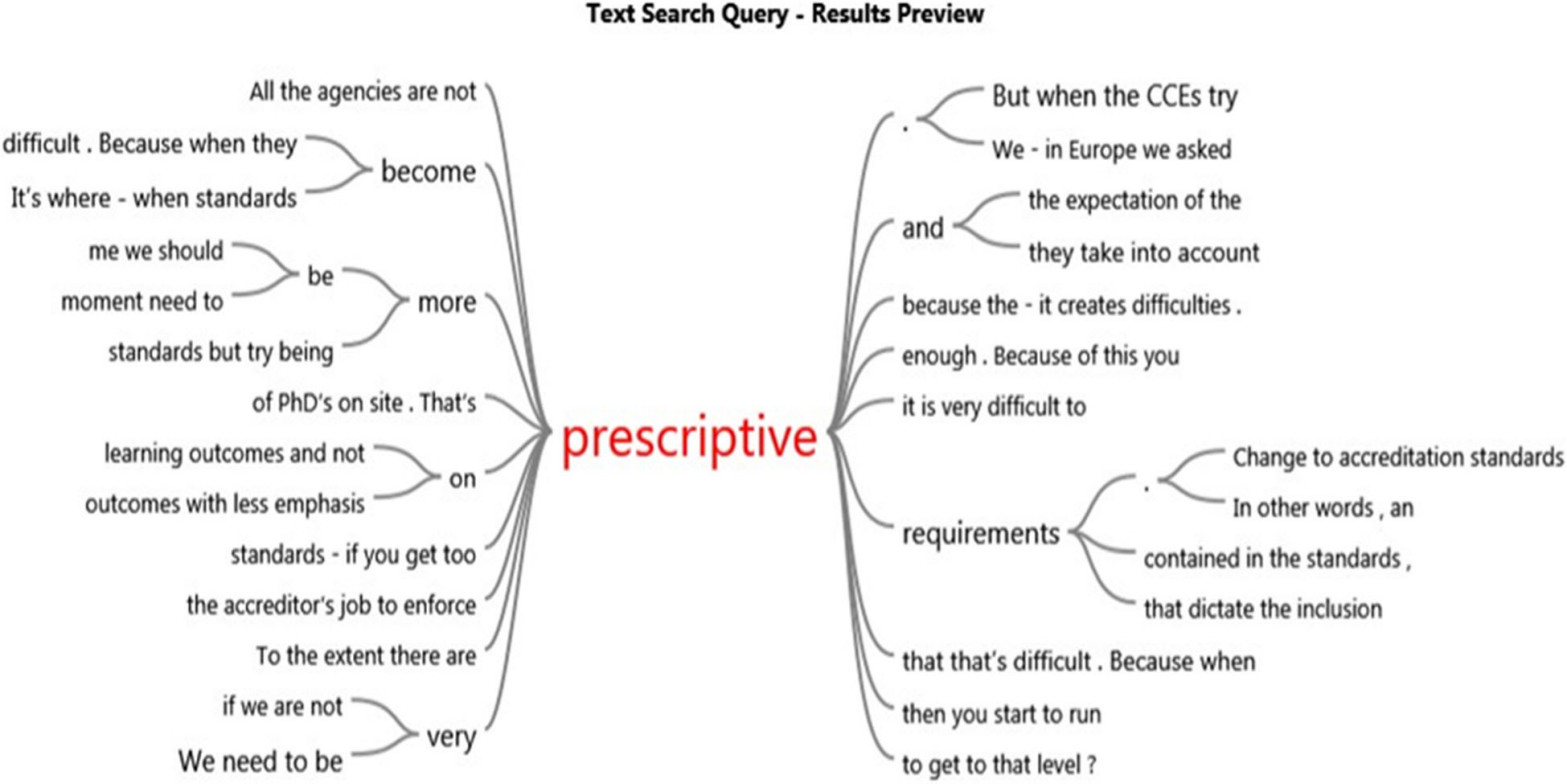
Prescriptive

**Fig. 3:**
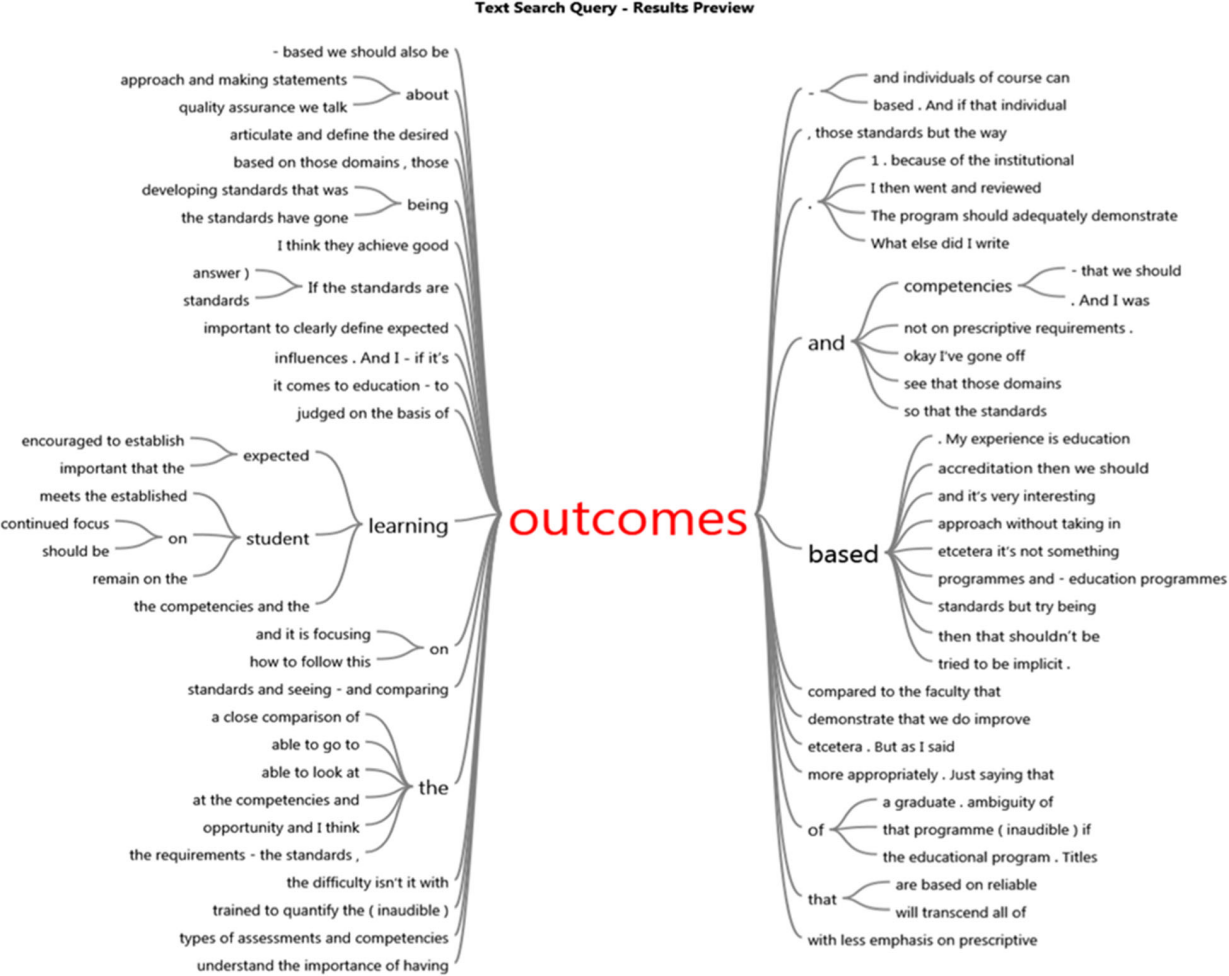
Outcomes

**Fig. 4:**
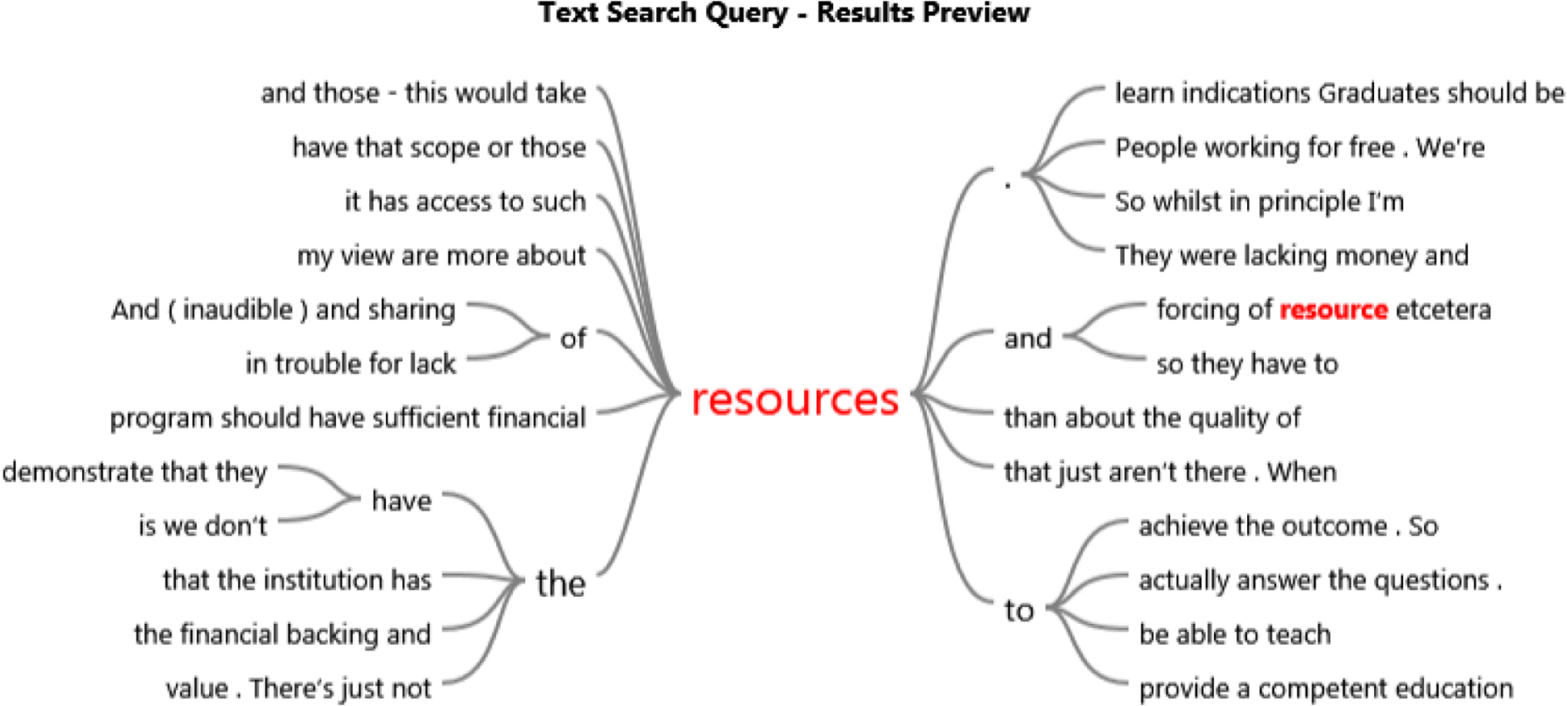
Resources

In support of these themes, word trees developed from the NVivo software are presented in Figs. 2, 3, and 4. The word trees were developed from the verbatim quotes of the participants. To illustrate these ‘word trees’ the responses for the themes “to focus on outcomes or be prescriptive?” and “more resources please” are diagrammatically captured as examples.

#### Theme 1: professional differences

The dominant recurring theme from all respondents across the questions concerned the difficulties CCEs encounter when carrying out their functions as a result of various and diverse interest groups’ strongly held opinions. This was mentioned in the context of establishing accreditation and competency standards as well as when defining terms such as ‘chiropractic’ and ‘diagnosis’. Respondents thought that these vocal interest groups had little or no expertise in matters of education but nonetheless adopted philosophical standpoints that caused conflict, and in turn, resulted in negotiated settlements on definitions and standards.

R1: “If we see improvement as moving ahead quickly then unity and uniformity has been a challenge because we - we spent a lot of time discussing issues that were either philosophical or issues that we had to do because of this … and …well basically we were being brought down by internal qualms and problems”.

#### Theme 2: keep it in the family

Two thirds of respondents commented that careful decisions were required by CCEs to make sure that chiropractic continued to maintain its ‘uniqueness’. Threats were believed to be non-chiropractors acting as members on CCEs or CP site inspection teams who may apply other health profession accreditation standards and processes that were not transferable to the education of chiropractors. Interestingly, one respondent thought this uniqueness (chiropractic manipulative skills) was threatened by increasing female numbers in the profession who are perceived to be less physically able, and thus less likely to use traditional manipulative skills and are more likely to adopt ‘low force’ techniques.

R7: “You can’t just bring people - experts, educational experts from other fields, put them together and they will do a good job because they won’t understand the nuances of what it is to be a Chiropractor and what it is to – what is a Chiropractic institution - what inherent problems can exist”.

#### Theme 3: to focus on outcomes or be prescriptive?

Participants expressed varied views on how best to go about their business of assessing CPs. Some participants lamented the absence of detailed descriptive and prescriptive standards. Inherent in this stance was an acute awareness that existing competencies for graduates and accreditation standards are set at a minimum level. Others saw the way forward as focusing on student learning outcomes that demonstrate competency. However, this was tempered by a recognition that there is a paucity of research and evidence for such outcome measures.

R1: “I recommend continued focus on student learning outcomes with less emphasis on prescriptive requirements*”.*

R5: “I think it is a good idea (outcomes –based assessment) but we tend to only set the standards at the minimum. I think we need more. We need to be very prescriptive*.*”

The word trees (Figs. 2 and 3) developed from the verbatim quotes of transcripts from the participants, illustrate the context of ‘prescriptive’ and ‘outcomes’ in the interview transcripts and provides perspective of the CCE respondents thinking. These Word Tree displays are produced by the Nvivo software as a graphic representation of the results of a text search query. They represent the context in which the word occurs.

#### Theme 4: more resources please

All but one of the respondents commented that the questions raised in the interviews were relevant but could not be dealt with by CCEs because they were run voluntarily by chiropractors without expertise or were under-resourced. Resources most commonly identified were financial in nature and targeted at research for improving the quality of CCE assessments, interventions and staff / member training. The word tree, Fig. 4, also developed from the verbatim quotes of transcripts from the participants, illustrates the context of “Resources” in the interview transcripts and also provides perspective of the CCE respondents thinking.

R4: “And another one (way to improve quality of CCEs) is from a different angle is CCEs if they had unlimited funds or - you know sort of generous funding they could also look to fund research and perhaps sponsor activities within a programme to run trials as to whether they would be helpful or otherwise”.

R6: *“*Just saying that they need to be valid assessments without giving them any guidance is challenging because most chiropractors who are in academia and CCEs do not have an education background. They’re practitioners. And you can’t expect that they’re going to understand and know that or even the managers. And I think it’s kind of obvious that they don’t”.

#### Theme 5: inter professional integration

Seven of the nine respondents thought that chiropractic practice was too isolated and needed to be more integrated into mainstream healthcare. Chiropractic graduates were thought to require a greater capacity than that was currently being demonstrated by practicing chiropractors to communicate more effectively with other healthcare professionals, with the perceived end result being improved patient care.

R1: “So inter-professional skills - you know as much as they can be put into standards or part of curriculums (sic) there’s almost a complete need to be there because we - we’re not good at this, we’re not good at having conversations with other professionals. We’re not good at relating to other professionals. As a profession we are somewhat paranoid”.

#### Theme 6: CPs making ends meet

The necessity for a CP to remain financially viable was mentioned by several respondents as a possible factor in several related issues that CCEs encounter. This included; CPs inappropriately lowering student admission requirements, confidentiality of accreditation processes being used to avoid CP brand damage when they perform poorly thus potentially negatively impacting on their enrolling student numbers, a motivator of unethical student practice behaviours on graduation, and a difficulty for CPs that are part of a university trying to obtain adequate funding.

R8: “And it’s a motherhood statement - a major sort of a motherhood statement I guess I think institutions are driven by motivations other than producing the highest quality graduates. I think some of the motivations have more to do with bums on seats and the amount of money that they can generate.”

## Discussion

### Overview

This is the first study to explore CCE views on accreditation standards and processes of CPs. Six themes were found throughout the semi-structured interviews. The respondents thought that CCE activities frequently involved having to negotiate diverse and strongly held differences in opinion from professional groups. The education of students about chiropractic practice was thought to need to become more interdisciplinary in nature while doing so without losing the ‘uniqueness’ of chiropractic. Respondents were diverse in their views on how CCEs should go about their business of accrediting CPs and improving the quality of chiropractic graduates. Finally, CCEs were viewed as being without sufficient resources to undertake their roles and thought that financial drivers were important motives for poor CP performance.

#### Theme 1: professional differences

The current accreditation standards, according to many of the CCE experts in this study, were a result of a negotiated settlement between disparate interest groups (vitalists versus evidence-based) and resulted in standards that allow all chiropractic views to co-exist. Some respondents in this study, in accord with the educational position statement, have advocated for the removal of *vitalism* from CPs [48]. Also respondents in this study are in accord with the recent World Federation of Chiropractic Education consensus statement calling for the support of an evidence-based teaching and learning based environment as the way forward for chiropractic education [49]. Moreover research of medical accreditation is demonstrating that accreditation standards need to begin with a review of the evidence-base for each standard [50]. ‘Unorthodox’ views have direct implications for patient safety and quality of care, for example anti-vaccination beliefs and the non-guideline use of X-rays [32]. We contend that the primary considerations by CCEs should be for patient safety, efficiency and quality of care and not a careful negotiation between past and present concepts.

In our opinion, negotiated settlements on accreditation standards or silence by CCEs that are driven by inclusive considerations are untenable if chiropractic is to become an integrated respected mainstream healthcare health profession. Chiropractic education delivered by CPs, and accredited by CCEs, must whole heartedly adopt patient-centred and evidence based drivers.

#### Theme 2: keep it in the family

The view by the CCE experts in this study that only chiropractors should be involved in CCEs for the establishing, monitoring and decision making of accreditation standards and processes of chiropractic education is challenged by studies in other healthcare professions [51, 52]. In a recent systematic review, only modest evidence could be found to support the importance for including medical doctors in the composition of governing bodies of healthcare organisations and hospitals [53]. Studies included in this review found that higher levels of performance was found in those medical doctors who were dedicated to their organisational role, had further management training and were not in part-time practice at the same time. Another included study found that low performance was most obvious in organisations where there was a low number of senior medical consultants that exerted a disproportionate influence over organisational priorities [53]. It is possible that there are similarities between medical practitioners and chiropractors when they are trained as health practitioners and then become involved in managing general organisational–operational business performance.

These findings suggest that CCEs may be well served to recruit appropriately trained full-time managers, who are not necessarily trained as chiropractors, but rather have strong management and strategizing skills. The potential upside is considerable, as it would provide CCE with people who can manage disparate voices and plan and organise complex and difficult strategies, such as the implementation of an evidence-based approach. In addition, this evidence suggests there would be a greater potential for promoting interdisciplinary interaction.

Alternatively chiropractors who work for CCEs could be funded to pursue advanced degrees in areas such as adult education theory and pedagogy. This approach has the possible benefit of allaying concerns about losing the ‘uniqueness’ of chiropractic by not requiring non-chiropractors to be in oversight roles. Ultimately however, research is required to explore how well chiropractors manage expected operational and management roles in CCEs is required to confirm whether or not these assumptions of similarities can be drawn with medical practitioners in organisations. Some CCEs have had full-time staff for many years, sometimes non-chiropractors, and these could be a rich source of data for further qualitative inquiry.

#### Theme 3: focus on outcomes or be prescriptive?

Most respondents’ views in this study were in accord with the international trend in medical education to move toward competency-based education [33, 54–56]. Purported benefits are a shared set of expectations around a common descriptive language for education that increases accountability for stakeholders [55, 57] and new avenues to assess overarching competencies, such as communication [56]. However, some respondents raised concerns, also expressed in the medical accreditation literature, that there is considerable heterogeneity in how the outcomes are defined, developed, implemented and assessed [54, 58, 59]. Consequently, some accreditation researchers have argued for a ‘hybrid’ model that contains both prescriptive detail and an outcomes-based approach [60]. This was also the case for some respondents in this study who expressed a desire for the retention of minimum numbers. For example, the number of patient treatments before graduation.

A hybrid system raises at least two important questions. First, what is chiropractic and its attendant scope of practice and second, what is the best model to deliver the most relevant education for those seeking to become chiropractors in the twenty-first century of healthcare? For example, should a chiropractic curriculum include courses on x-ray physics and positioning in the twenty-first century when the minority of chiropractors purchase x-ray equipment and clinical practice guidelines do not recommend the routine use of x-rays? Also some geographic regions have their curriculum content determined by requirements beyond the CCE alone. For example, the CPs in the USA and Canada need to prepare students to pass National Board examinations in subjects that some would deem irrelevant or inconsistent with the research evidence such as microbiology, histology and embryology [61].

These issues present a number of complex challenges for CCEs. The proffered pursuit of advanced education for CCE members and drawing on expertise from other accrediting bodies may go some way to assisting in this journey. Several studies suggest that the creation of language definitions (for example “chiropractor”) is critical to research and development of a set of equivalent evidence-based accreditation standards and processes that will be educationally useful [62, 63] and indeed this may be a starting point.

#### Theme 4: more resources please

This results of this study suggests that CCEs are staffed by chiropractors who have, and continue to, generously provide time to voluntarily participate with the intention of improving the quality of chiropractic care. Consideration needs to be given to ways to appropriately fund CCEs. This will likely require differing strategies for each CCE region and further qualitative interviews with participants from CCEs, CPs, professional associations and other regulatory or government agencies may generate possibilities. With the right level of resources CCEs could, among others, employ experts from allied healthcare disciplines, deliver training for executives in leadership and fund advanced degrees in education for CCE members for decision making about accreditation standards for the twenty-first Century. Also it could fund training for financial, management and organisational strategy skills, site inspection teams, and fund research for improved language clarity in their accreditation standards. It would also allow CCEs to be composed of larger numbers of skilled executives and avoid the accusation of low numbers of senior members with a disproportionate influence over CCE priorities [53].

#### Theme 5: inter-professional integration

Respondents spoke of the need for increased interdisciplinarity for chiropractic education and practice. This is difficult for many CPs as the educative process predominately takes place in private colleges and they are not exposed other health professionals in the classroom or clinical setting. There are successful examples in Denmark and Switzerland where this has taken place and this appears to be facilitating the integration of chiropractic into mainstream healthcare [64].

Research has identified barriers, albeit in practicing chiropractors, to improving inter-professional relations and this includes practitioners perceiving that their treatment model is preferable to biomedical alternatives [18]. Another factor seems to be a mindset that chiropractic is “unique” or “separate and distinct” [65]. Social Identity theory explains this as an ‘us versus them’ mindset [66, 67]. There are several educative possibilities that may assist in challenging this instinctive thinking. For CPs the obvious, but often expensive and time intensive option, is hospital placements for chiropractic students. Another possibility may be to form a collaboration with other universities or colleges that offer multiple health professional training programs and require students from differing disciplines to combine to undertake a common assessment task or project (either face-to-face or on-line).

CCEs may wish to further explore options by opening dialogues with other allied-health or medical education accreditation agencies to explore ways to facilitate interdisciplinary communication [31, 68, 69]. Often allied and medical accrediting agencies have expended large amounts of time, money, research and expertise refining their standards and process for accreditation [29].

#### Theme 6: CPs making ends meet

Many CPs are private programs (especially in the U.S.A.) and are tuition dependent. According to the respondents in this study, this appears to create pressure on them to relax admission standards in order to remain financially viable. CCEs control over this issue is limited to setting standards of educational excellence for training clinically competent chiropractors.

Financial concerns as generators for poor behaviour of education providers is not restricted to CPs. Aggressive and potentially misleading recruitment practices, poor ethical practices, and inappropriate commercial influences have been documented in other health education programs [70, 71]. Dialogue with other health professional education accreditation agencies who have successfully managed this issue may be useful.

It is curious that CCE respondents did not raise the issue of high student loan debts and default rates. This appears to especially problematic in the U.S.A [72]. Here, students have been shown to demonstrate inadequate financial literacy [72]. CCEs may have a role to play in ensuring the curricula of CPs are appropriate and adequate to address this literacy concern.

### Strengths and limitations

This study sampled the views of nine experienced CCE past and present members with an average of 14 years’ experience and a response rate of nearly 70%. We were not able to compare responders to those who refused to participate (non-responders). Several participants had experience on a number of CCEs. We are confident they have provided a rich insight into the issues surrounding CCEs. However, as this was a qualitative study, our sample cannot be assumed to be representative of the views of all members of all CCEs internationally and could be infleuenced by community bias. The authors are confident they have addressed the issues surrounding qualitative research of reflexivity [73], credibility, transferability, dependability and confirmability [47].

### Recommendations

This study has led to the identification of a number of issues and, based on these as well as the available literature, the authors make a number of individual recommendations that are summarised in Table 2. If these recommendations are adopted, then outcomes such as a uniform high standard of practitioners who are evidence-based and lifelong learners is likely to be enhanced across all CCE-controlled regions. This would help ensure and safeguard the international trust in practitioners’ ability to deliver ethical, safe and quality care across world-wide borders.

**Table 2:** Summary table of recommendations for CCEs

	Recommendation	Justification
1	Internationally uniform definitions of basic terms such as chiropractic, diagnosis, and scope of practice are required.	Uniform and high quality methods of assessment for student learning-outcomes, and site inspection reports can be created to create standardised assessment of CPs across CCEs. Common standards would ensure and safeguard patient safety and care and be good for global workforce standardisation.
2	Use acquired definition and scope of practice for the creation of reliable and valid measures for assessing student learning.	Uniform assessment of CPs can allow for more accurate baseline measures from which quality improvements can be monitored.
3	Funding sources be identified for CCEs.	This would allow CCEs to conduct their own quality improvements such as staff training and employ highly qualified people without a conflict of interest
4	CCE executives should ideally be full-time.	Part-time practice and part-time organisational involvement leads to poorer executive performance levels.
5	CCEs composition should include non-chiropractors with managerial and organisational strategy skills.	This would provide CCEs with skill sets to manage the varied professional interest groups, establish standardised training for members and site inspections, develop strategies to increase CP compliance, and have a greater potential for promoting interdisciplinary.
6	CCEs should consider specialised further education for their executive members relevant to their roles.	As above.
7	Facilitate research that explores an outcomes-based and prescriptive approach to the competency levels of graduating chiropractic students. For example, the number of classroom hours, the number of patient treatments.	This will develop, inform and improve regulatory standards.
8	Actively regulate and remove Vitalism and ‘subluxation’ from CP curricula unless it is taught in a historical context.	Align chiropractic education with contemporary evidence-based approaches to health profession education.
9	Engage with other health disciplines education accreditation bodies.	Gain expertise and research for quality improvement of accreditation standards and processes
10	Adoption of a patient-centred approach to accreditation standards and processes	Align with contemporary mainstream healthcare.
11	Adopt an evidence-based approach to accreditation standards and processes	Align with contemporary mainstream healthcare.
12	A review of the chiropractic curriculum to remove or streamline outdated courses. For example radiography, histology, and embryology.	To better align chiropractic education with twenty-first Century healthcare.

## Conclusions

The aim of this study was to report on key informant opinions of Councils on Chiropractic Education (CCE) regarding recent research findings reporting on improving accreditation standards and processes for chiropractic programs.

To this end, respondents were asked their views on a number of issues arising from these recent studies that have identified issues and possible explanations. Six themes were found across the semi-structured questions.

Diverse professional interest groups were viewed as creating considerable conflict within CCEs and these conflicts were pivotal in the formation of negotiated accreditation standards. There is a lack of clarity of important and basic terms such as “chiropractor” and the resultant scope of practice. This in turn has partially resulted in the creation of accreditation standards and processes that allow for the co-existence of evidence-based/evidence-friendly and philosophy driven CPs. This situation is further reinforced by a feeling that there is a ‘uniqueness’ of chiropractic practice that must be preserved and a lack of financial and personnel resources for CCEs to adequately conduct their business.

Recommendations are made to improve the standards and process of CCEs for (re)-accreditation of CPs, including a widespread adoption of evidence-based approach and changing the focus of CCE accreditation standards to serve the public and patients and not the profession.

## Additional file

Additional file 1: Aide de Memoire / Interview questions. (DOCX 17 kb).

## Data Availability

Not applicable.

## Abbreviations

CCE: Council on Chiropractic Education
CP: Chiropractic Program
